# Fc****γ****-Receptor IIIA Polymorphism p.158F Has No Negative Predictive Impact on Rituximab Therapy with and without Sequential Chemotherapy in CD20-Positive Posttransplant Lymphoproliferative Disorder

**DOI:** 10.1155/2014/264723

**Published:** 2014-02-10

**Authors:** Heiner Zimmermann, Theresa Weiland, Jamie P. Nourse, Maher K. Gandhi, Petra Reinke, Ruth Neuhaus, Mohsen Karbasiyan, Barbara Gärtner, Ioannis Anagnostopoulos, Hanno Riess, Ralf U. Trappe, Stephan Oertel

**Affiliations:** ^1^Department of Internal Medicine II: Hematology and Oncology, University Medical Center Schleswig-Holstein, Campus Kiel, 24105 Kiel, Germany; ^2^Department of Hematology, Charite University Hospitals, Campus Virchow-Klinikum, 13353 Berlin, Germany; ^3^Clinical Immunohaematology Laboratory, Queensland Institute of Medical Research, Brisbane QLD 4006, Australia; ^4^Department of Nephrology and Intensive Care, Charité-Universitätsmedizin Berlin, Campus Virchow-Klinikum, 13353 Berlin, Germany; ^5^Department of Abdominal and Transplant Surgery, Charité-Universitätsmedizin Berlin, Campus Virchow-Klinikum, 13353 Berlin, Germany; ^6^Department of Medical Genetics, Charité-Universitätsmedizin Berlin, Campus Virchow-Klinikum, 13353 Berlin, Germany; ^7^Department of Microbiology, Saarland University Hospital, 66421 Homburg, Germany; ^8^Department of Pathology, Charité-Universitätsmedizin Berlin, Campus Mitte, 10117 Berlin, Germany; ^9^Global Medical Affairs Oncology, F. Hoffmann-La Roche Ltd., 4070 Basel, Switzerland

## Abstract

We retrospectively analyzed the p.V158F polymorphism of Fc**γ**-receptor IIIA (FCGR3A, CD16) in patients with PTLD treated with rituximab monotherapy. Previous reports had indicated that the lower affinity F allele affects rituximab-mediated antibody-dependent cellular cytotoxicity (ADCC) and is linked to inferior outcome of rituximab monotherapy in B cell malignancies. 25 patients with PTLD after solid organ transplantation were included in this analysis. They had received 4 weekly doses of rituximab as part of two clinical trials, which had a rituximab monotherapy induction regimen in common. 16/25 patients received further treatment with CHOP-21 after rituximab monotherapy (PTLD-1, NCT01458548). The FCGR3A status was correlated to the response after 4 cycles of rituximab monotherapy. Response to rituximab monotherapy was not affected by F carrier status. This is in contrast to previous findings in B cell malignancies where investigators found a predictive impact of FCGR3A status on outcome to rituximab monotherapy. One explanation for this finding could be that ADCC is impaired in transplant recipients receiving immunosuppression. These results suggest that carrying a FCRG3A F allele does not negatively affect rituximab therapy in immunosuppressed patients.

## 1. Introduction 

Posttransplant lymphoproliferative disorder (PTLD), a spectrum of lymphatic diseases associated with the use of potent immunosuppressive drugs after transplantation [1], ranges from polyclonal early lesions associated with primary EBV infection to monomorphic lymphoma [2]. The value of rituximab therapy in CD20-positive PTLD has been established by prospective, phase II trials of first-line rituximab monotherapy [3–5]. The PTLD-1 trial, the largest prospective phase II trial in the field so far, has demonstrated the efficacy and safety of sequential therapy (rituximab followed by CHOP chemotherapy) with an overall response rate of 90% and 6.6 years median overall survival. However, the outcome of rituximab nonresponders was significantly poorer than that of responders [6].

Factors contributing to interindividual variability in the response to rituximab are therefore of therapeutic relevance and have recently been reviewed [7]. They include tumor-related factors as well as host-related factors linked to the suggested mechanisms of action of the chimeric monoclonal anti-CD20 antibody rituximab: direct cytotoxicity [8], complement-dependent cytotoxicity (CDC) [9], and antibody-dependent cellular cytotoxicity (ADCC) [10].

Of particular interest among the host factors is the polymorphism G559T (p.V158F) of* FCGR3A*, which encodes Fc*γ*RIIIA (CD16), a low-affinity receptor for the Fc fragment of IgG, on immune effector cells [11]. The phenylalanine (F) allele has a lower affinity for human IgG1 [11] and affects rituximab-mediated ADCC [12]. In follicular lymphoma, carrying one or two Fc*γ*RIIIA p.158 F alleles was associated with a poorer response to [13, 14] and shorter time to progression (TTP) [14] after rituximab monotherapy. Data from 72 patients treated with chemotherapy and either rituximab or I-131 tositumumab in SWOG trials suggested an association between carrying at least one Fc*γ*RIIIA p.158 V allele and better overall survival [15]. However, there was no difference in response rate or outcome in follicular lymphoma treated with rituximab-based immunochemotherapy in a large subpopulation (*n* = 460) of the PRIMA trial [16]. In high-grade lymphomas, a multivariate analysis of patients with diffuse large B cell lymphoma treated with R-CHOP in the RICOVER-60 trial identified a nonsignificant trend towards an unfavorable outcome for carriers of Fc*γ*RIIIA p.158 F/F [17].

PTLD is defined by the patients' impaired cellular immune response due to immunosuppression. ADCC in particular has been shown to be impaired in transplant recipients receiving immunosuppression such as azathioprine (AZA) and steroids [18–20] or cyclosporine A (CyA) and steroids [21]. *In vitro* treatment of human NK cells with CyA, rapamycin, mycophenolate (MMF), and FK506 has led to reduced NK-cell cytotoxicity [22–24]. We therefore set out to analyze the impact of the Fc*γ*RIIIA p.158 polymorphism on interindividual variability of treatment response in patients with CD20-positive PTLD treated with rituximab monotherapy in prospective trials [3, 6].

## 2. Materials and Methods

### 2.1. Patients

9/25 patients were enrolled in the German phase II trial investigating rituximab monotherapy in CD20-positive PTLD [3] and 16/25 patients were enrolled in the European Study Groups on PTLD prospective phase II trial investigating sequential treatment with rituximab and CHOP-21 in CD20-positive B cell PTLD (PTLD-1, NCT01458548) [6]. This analysis focuses on the responses seen after 4 cycles of rituximab monotherapy. Patients were selected for availability of peripheral blood samples for Fc*γ*RIIIA p.158 genotyping—in the majority patients treated at Charité, Berlin. All patients provided written informed consent. The diagnosis of PTLD was reviewed by an expert pathologist (IA) according to the WHO classification. Treatment consisted of four weekly applications of rituximab in both trials. Further details on the trials can be found in the respective publications [3, 6].

### 2.2. Controls

One hundred and five consenting healthy adult Caucasian volunteers served as controls as previously published [25, 26]. Controls specifically denied hematological or autoimmune disorders of any kind. Study of healthy controls was approved by the Queensland Institute of Medical Research Ethics Committee and signed informed consent was obtained from all participants.

### 2.3. DNA Isolation

Automated purification of genomic DNA was performed from 230–350 *μ*L peripheral blood samples using an automated workstation and the QIAGEN Mag Attract kit (Qiagen, Hilden, Germany) according to the manufacturers' instructions.

### 2.4. Fc*γ*RIIIA Genotyping

**Figure 1:**
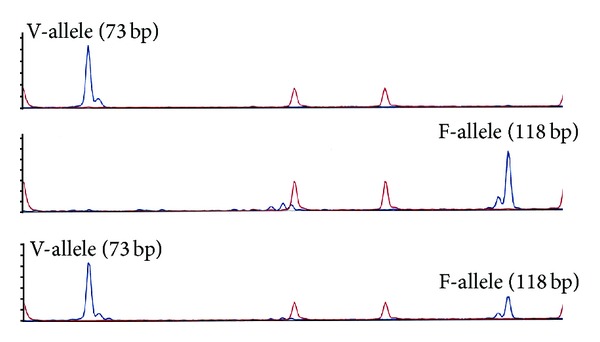
Fc*γ*RIIIA polymorphism genotyping. Upper line: patient homozygous for the Fc*γ*RIIIA p.158 V-allele, 2nd line: patient homozygous for the Fc*γ*RIIIA p.158 F-allele, lower line: heterozygous for the Fc*γ*RIIIA p.158 V and F-allele.

FCGR3A G559T (p.V158F, dbSNP rs396991) genotyping was performed by allele-specific PCR as previously published [25], based on the method described by Dall'Ozzo et al. [27] using the following primers: common forward: 6FAM-5′-TCCAAAAGCCACACTCAAAGTC-3′, F-allele-specific reverse: 5′-GCGGGCAGGGCGGCGGGGGCGGGGCCGGTGATGTTCACAGTCTCGTAAGACACATTTTTACTCCCAGA-3′, and V-allele-specific reverse: 5′-TGAAGACACATTTTTACTCCCATC-3′. To achieve specific amplification of FCGR3A despite the high homology to FCGR3B the forward primer is placed with the 3′ base specific to the 3A gene sequence. To further increase specificity the 3′-1 base is deliberately mismatched. Reverse primers are designed to be specific to the polymorphism and also have 3′-1 mismatches for specificity. To allow discrimination between the two allelic PCR products a 26 bp 5′ extension is added to the F allele primer, and to prevent the V primer extending using the F primer as template a mismatch is incorporated in the F primer corresponding to the two 3′ bases of the V primer. Reagent concentrations were 0.2 *μ*mol/L common forward and V-allele-specific reverse primer, 0.1 *μ*mol/L F-allele-specific reverse primer, 0.4 *μ*L AccuPrime Taq DNA polymerase (Invitrogen), 2 *μ*L AccuPrime buffer I, 1 mmol/L MgCl_2_, and 50 ng genomic DNA. Reaction volume was 20 *μ*L. PCR cycling conditions were 2 min denaturation at 94°C followed by 35 cycles (94°C for 15 s and 60°C for 15 s). Resulting products were diluted (1 : 20) and 1 *μ*L of this dilution was analyzed on a 48-capillary DNA analyzer (ABI 3730, Applied Biosystems, Foster City, USA) using the GeneScan 400 HD ROX size standard for sizing DNA fragments in the 50–400 nucleotides range. The method has been validated by direct sequencing of selected samples as previously reported [25]. Patients homozygote for FCGR3A 559G (Fc*γ*RIIIA p.158V) alleles were counted as non-F-carriers whereas heterozygotes and those homozygote for 559T (Fc*γ*RIIIA p.158F) were counted as F carriers. Representative examples for VV, FF, and VF genotypes are shown in Figure 1.

### 2.5. Data Analysis

Exploratory univariate analyses were performed by applying *χ* ^2^ tests to categorical variables. The level of significance in all cases was set at *P* < 0.05. Statistical tests were performed using IBM SPSS 20 and GraphPad Prism 5.03.

## 3. Results

Peripheral blood samples for genotyping were available from a total of 25 out of 87 patients treated in the two clinical trials. Importantly, the patients evaluated for the Fc*γ*RIIIA p.158 polymorphism were representative subsets of the trial populations they were initially treated in. The baseline characteristics of 25 patients are summarized in Table 1. The two groups of F and non-F-carriers are balanced regarding PTLD-specific disease characteristics like transplant type, time to transplantation, histology, and EBV association, as well as for age, gender, disease stage, and performance status.

### 3.1. Fc*γ*RIIIA p.158 Polymorphism

Of the 25 patients investigated for Fc*γ*RIIIA p.158 polymorphism 11 were non-F-carrier and 14 F carrier. Of the control patients 9 were non-F-carrier and 96 F carrier. The frequency distribution of Fc*γ*RIIIA p.158 genotypes in the control and patient groups both were consistent with a Hardy-Weinberg equilibrium. However, the distributions were significantly different (*P* < 0.0001) with non-F-carriers (Fc*γ*RIIIA p.158V homozygotes) being much more frequent in the PTLD group compared to healthy controls (Table 2).

### 3.2. Fc*γ*RIIIA p.158 Polymorphism and Immunosuppression

In the PTLD-1 sequential therapy trial detailed information on immunosuppression is available. Pre-PTLD regimens and doses of immunosuppression were similar with respect to mTOR, calcineurin inhibitor (CNI), and antimetabolite (azathioprine and MMF) use, but non-F-carriers more frequently received low-dose steroids (*P* = 0.05, Table 3). After diagnosis of PTLD, CNIs were maintained in both groups but doses were reduced by 20 to 50%. Patients with high affinity Fc*γ*RIIIA were maintained on steroid-based immunosuppression and stopped antimetabolites, specifically MMF, resulting in dual immunosuppression in 7/8 cases. Patients with low affinity Fc*γ*RIIIA, however, frequently were maintained on antimetabolite containing immunosuppression and only the patient receiving azathioprine was switched to steroids.

### 3.3. Fc*γ*RIIIA p.158 Polymorphism and Treatment Response

8/11 non-F-carriers and 9/14 F carriers had at least a partial response after 4 cycles of rituximab monotherapy. Thus, there was no statistically significant difference by F carrier status in overall response to rituximab monotherapy (*P* = 0.65). There were also no statistical significant differences with respect to complete and partial response by F carrier status (*P* = 0.35 and 0.55), respectively (Table 4).

## 4. Discussion

We present a group of patients with PTLD, who were treated within prospective trials [3, 6]. Limitations are the small overall number of patients and the retrospective analysis of a subgroup of patients from clinical trials. The Fc*γ*RIIIA p.158 polymorphism did not affect the response to rituximab monotherapy in the 25 evaluated patients with PTLD. Thus, carrying a low affinity Fc*γ*RIIIA p.158F allele was not associated with a poorer treatment response and efficacy. In this respect, our results are in contrast to the findings for rituximab monotherapy in F carriers in immunocompetent patients with FL [13, 14] and could suggest a limited role of rituximab-mediated ADCC in the treatment of PTLD. However, rituximab monotherapy is a very efficacious treatment in PTLD. So, the general question may be raised on the role of ADCC as a major mode of action for cell killing in the immunosuppressed individual.

We have also observed a significantly higher frequency of the Fc*γ*RIIIA p.158V homozygote (i.e., high-affinity) genotype in solid organ transplant recipients with PTLD compared to healthy controls. We speculate that the immunosuppression in patients diagnosed with PTLD as the major difference to B cell lymphomas in the immunocompetent setting is the reason for the observed distribution differences in F carrier status. At first glance these results are incongruent with a previous, larger study by Stern et al. [28], which reported no association of Fc*γ*RIIIA p.158 genotype with the risk of PTLD development. While the PTLD patients reported here were in the majority advanced stage, monomorphic PTLD that had not responded to immunosuppression reduction, the majority of patients analyzed by Stern et al. had localized disease, histology was not analyzed, and a proportion of patients was treated before 1997, when rituximab was not available. While treatment was not recorded, standard treatment for localized PTLD then was immunosuppression reduction ± local therapy. The overall survival outcome was favorable, suggesting a good response to immunosuppression reduction, in contrast to the patients analyzed here. 

Establishing associations between candidate genes and PTLD susceptibility may provide insight into pathogenesis and support the development of therapeutic strategies. However, it is imperative for studies on genetic susceptibility to assign patients definitively to specific PTLD phenotypes. PTLD on the other hand is a heterogeneous disease ranging from closely EBV-related early lesions to polymorphic PTLD to aggressive, monomorphic lymphomas, where EBV association is less frequent. While immunosuppression is high in patients with PTLD diagnosed within the first year after solid organ transplantation, late PTLD results from a more chronic immunosuppression at lower doses. The relation of immunosuppression to Fc*γ*RIIIA p.158 genotype and PTLD development thus might be complex.

Our observation that solid organ transplant recipients with PTLD carrying a Fc*γ*RIIIA p.158V allele more often had triple immunosuppression including low-dose steroids compared to individuals with a Fc*γ*RIIIA p.158F homozygote (i.e., low-affinity) genotype might help to explain the distribution difference. Carrying a Fc*γ*RIIIA p.158 VV high-affinity genotype might result in a more frequent need for steroid use or a more potent immunosuppression in these transplant recipients, thus influencing an individual's risk for PTLD development. However, further studies are warranted to analyze the association of Fc*γ*RIIIA p.158 genotype, immunosuppression, and PTLD in more detail.

In summary, our findings on the effect of the Fc*γ*RIIIA p.159 polymorphism in PTLD suggest a genetic risk factor for PTLD, mediated through higher doses of immunosuppression; a reduced role of ADCC as a rituximab effector mechanism in the posttransplant setting; and competition between ADCC and other rituximab effector mechanisms *in vivo*.

Regarding PTLD, the apparent lack of efficient ADCC in PTLD provides a rationale for continuing rituximab treatment beyond 4 applications so that immunosuppression reduction has more time to take effect and the competition between ADCC and other rituximab effector mechanisms can be overcome by higher rituximab levels (i.e., R-CHOP immunochemotherapy instead of CHOP chemotherapy after rituximab monotherapy). The validity of this approach will be assessed by data from our risk-stratified sequential treatment (RSST) trial in PTLD (PTLD-1, 3rd amendment, NCT00590447), which will be mature in 2014. In addition, second-generation monoclonal anti-CD20 antibodies such as GA101 [29], which promise stronger ADCC but less CDC, will have to be carefully evaluated for their benefit in the setting of PTLD.

## Figures and Tables

**Table 1:** Patient's baseline characteristics.

	Fc*γ*RIIIA p.158 evaluated	Fc*γ*RIIIA p.158 non-F-carrier	Fc*γ*RIIIA p.158 F carrier	*P*
Number of patients	25	11	14	
Age/years: median (range)	51 (18–74)	51 (18–74)	48 (23–68)	n.s^†^
Male	17/25	8/11	9/14	n.s.
Transplant type				
Kidney	11/25	6/11	5/14	n.s.
Liver	7/25	2/11	5/14	
Heart	6/25	3/11	3/14	
Kidney + pancreas	1/25	0/11	1/14	
Time from transplantation to PTLD/years: median (range)	3.9 (0.4–25.3)	3.7 (0.4–25.3)	6.1 (0.4–15.4)	n.s^†^
More than 1 year	20/25	9/11	11/14	n.s
Histology				
Polymorphic	2/25	1/11	1/14	n.s.
Monomorphic	23/25	10/11	13/14	
Burkitt	1/23	0/10	1/13	
DLBCL	17/23	7/10	10/13	
Other B cell	5/23	3/10	2/13	
EBV association	12/25	6/11	6/14	n.s.
Ann Arbor stage				
I/II	6/25	2/11	4/14	n.s.
III/IV	19/25	9/11	10/14	
LDH (upper limit of normal 240 U/L)				
Elevated*	13/24*	8/11	5/13*	n.s.
Disease manifestations				
Nodal disease	15/25	5/11	10/14	n.s.
Extranodal disease	20/25	10/11	10/14	n.s.
ECOG performance status				
<2	18/25	7/11	11/14	n.s.
≥2	7/25	4/11	3/14	

DLBCL indicates diffuse large B cell lymphoma; EBV: Epstein-Barr virus; and ECOG: Eastern Cooperative Oncology Group. *Parameter not evaluated in the missing patients; ^†^Mann-Whitney *U* test.

**Table 2:** Frequency of Fc*γ*RIIIA p.V158F genotypes and alleles in healthy controls and PTLD patients.

Genotype	Controls	PTLD	*P*
Fc*γ*RIIIA p.158 V/V homozygote	9 (8%)	11 (44%)	<0.0001
Fc*γ*RIIIA p.158 V/F heterozygote	50 (48%)	11 (44%)	
Fc*γ*RIIIA p.158 F/F homozygote	46 (44%)	3 (12%)	

**Table 3:** Immunosuppression of patients treated in the PTLD-1 trial by Fc*γ*RIIIA p.158V/F polymorphism: non-F-carrier versus F carrier.

	Fc*γ*RIIIA p.158 non-F-carrier	Fc*γ*RIIIA p.158 F carrier	*P*
Number of patients	8/16	8/16	

Immunosuppression at diagnosis of PTLD, *n*/*N* (mean in mg, range in mg)			
mTOR inhibitors	2/8 (2, 1–3)	1/8 (1)	n.s.
Calcineurin inhibitors	7/8	7/8	n.s.
Cyclosporin A	3/8 (193, 180–200)	2/8 (195, 190–200)	n.s.
FK506	4/8 (5, 2–9)	5/8 (6, 5–8)	n.s.
Antimetabolites	4/8	5/8	n.s.
Azathioprine	1/8 (75)	1/8 (75)	n.s.
MMF	3/8 (1333, 1000–2000)	4/8 (1625, 1000–2000)	n.s.
Steroids	6/8 (6, 4–10)	2/8 (13, 10–16)	0.05

**Table 4:** Treatment response by Fc*γ*RIIIA polymorphism p.V158F: non-F-carrier versus F carrier.

	Patients in trial	Fc*γ*RIIIA p.158 evaluated	Fc*γ*RIIIA p.158 non-F-carrier	Fc*γ*RIIIA p.158 F carrier	*P*
∑ Response to 4 cycles rituximab (PT-LPD1 and PTLD-1 interim staging combined)					
Number of patients	87	25	11	14	
ORR		17/25	8/11	9/14	0.65
CR		11/25	6/11	5/14	0.35
PR		6/25	2/11	4/14	0.55

ORR denotes overall response rate, CR: complete remission, PR: partial remission, SD: stable disease, and PD: progressive disease. *Missing patients could not be evaluated for response due to early death.
